# Reliability and validity of the Japanese version of Pain Disability Index

**DOI:** 10.1371/journal.pone.0274445

**Published:** 2022-09-12

**Authors:** Keiko Yamada, Akira Mibu, Sonora Kogo, Michael Sullivan, Tomohiko Nishigami

**Affiliations:** 1 Pain Medicine, Juntendo University Graduate School of Medicine, Tokyo, Japan; 2 Department of Anesthesiology and Pain Medicine, Juntendo University Faculty of Medicine, Tokyo, Japan; 3 Department of Psychology, McGill University, Montreal, Quebec, Canada; 4 Department of Physical Therapy, Konan Women’s University, Kobe, Hyogo, Japan; 5 Department of Rehabilitation, Tanabe Orthopedics, Osaka, Japan; 6 Department of Psychology, Concordia University, Montreal, Quebec, Canada; 7 Department of Physical Therapy, Prefectural University of Hiroshima, Mihara, Hiroshima, Japan; Universita degli Studi Europea di Roma, ITALY

## Abstract

This study evaluated the reliability and validity of a Japanese version of Pain Disability Index (PDI). Analyses were conducted on a 7-item version (PDI-J) and a 5-item (PDI-5-J version of the PDI). Using a web-based survey system, we recruited 300 individuals with chronic low back pain (lasting ≥3 months) and 300 individuals with chronic daily headache (lasting ≥15 days per month for 3 months) aged 20–64 years. Analyses revealed a one-factor with goodness-of-fit indices assessed by confirmatory factor analysis. For concurrent validity, we calculated Pearson’s correlation coefficients among the PDI-J, PDI-5-J, Pain Disability Assessment Scale, Pain numerical rating scale, and revised version of Short-Form McGill Pain Questionnaire. Internal consistency was evaluated by Cronbach’s α, and test–retest reliability was assessed with intraclass correlations (ICCs) in 100 of 600 participants a week after the first response. Both Japanese adaptations of the PDI demonstrated good concurrent validity and reliability (Cronbach’s α was 0.89 for PDI-J in chronic low back pain or chronic daily headache, and 0.94 and 0.93 for PDI-5-J in chronic low back pain and chronic daily headache, respectively). The PDI-J and PDI-5-J showed were highly correlated (r = 0.98). ICCs were 0.67 and 0.59 for the PDI-J and 0.59 and 0.63 for the PDI-5-J in chronic low back pain and chronic daily headache, respectively. In conclusion, these two PDI versions can be potentially used for evaluating pain-related interference with daily activities among the Japanese general population.

## Introduction

Self-report measures of disability play an important role in research and clinical practice with chronic pain patients. Self-report measures of disability provide a cost-effective means of assessing the life impact of chronic pain. A patient’s self-report of how pain interferes with participation in a range of activities of daily living can provide the clinician with important information to guide the development of an appropriate treatment plan. As well, the Initiative on Methods, Measurement, and Pain Assessment in Clinical Trials (IMMPACT) advocates the use of self-report measures of disability as part of a comprehensive assessment of the effects of treatments provided for individuals with chronic pain [1–4].

The Pain Disability Index (PDI) [5] is a widely used self-report measure of pain-related disability. On this measure, respondents rate the degree to which their pain condition interferes with participation in seven different areas of daily living: home, social, recreational, occupational, sexual, self-care and life support activities. Respondents rate their level of disability on an 11-point scale with the end points 0 (no disability) and 10 (total disability). Several investigations have provided support for the PDI as a reliable and valid measure of pain-related disability [5–8]. The PDI has been used to assess pain-related disability in a wide range of debilitating pain conditions including chronic musculoskeletal pain [9, 10], whiplash [11, 12], fibromyalgia [13, 14], neuropathic pain [15, 16], migraine [17], cancer [18, 19], osteoporosis [20], orofacial pain [21, 22], and postsurgical pain [23, 24].

In a previous study, we described the linguistic validation of a Japanese version of the PDI (PDI-J) [25]. We showed that the Japanese translation of PDI was correctly interpreted by patients as a measure of pain-related disability. However, some patients remarked that they were not comfortable answering items about disability for sexual activities and life support activities. This discomfort likely arises from Japanese cultural norms that consider sexual behavior to be a very private matter and a reluctance to admit to limitations that might be interpreted as an imposition on others. Therefore, we also aimed to address the validity of a 5-item version of the PDI-J (PDI-5-J) that excluded the items assessing disability for sexual behavior and life support activities.

The purpose of the present study was to demonstrate psychometric validity and reliability of the PDI-J and PDI-5-J among individuals with chronic pain. The study sample consisted of individuals who had chronic low back pain and chronic daily headache. Participants completed the PDI-J and PDI-J-5 as part of a Japanese Biopsychosocial Assessment of Pain (JBAP) project. Analyses addressed the internal consistency of both measures. We hypothesized that analyses would support the reliability and validity of the PDI-J and PDI-5-J as measures pain-related disability in Japanese population.

## Materials and methods

### Study design

Data were drawn from a web-based survey as one of the components of the JBAP project (DC-JBAP2020). The DC-JBAP2020 was designed to investigate the validity and reliability of several Japanese pain-related questionnaires, including the PDI-J and PDI-5-J. In October 2020, we extracted the data of 600 Japanese workers (300 workers with chronic low back pain and 300 workers with chronic daily headache) aged 20–64 years. These 600 workers were selected from 5,000 responders among 12,500 candidates who received an e-mail invitation after being randomly selected from 28,117 panelists (12,521 panelists with low back pain and 15,596 panelists with headache) who registered through a Japanese internet survey agency (Rakuten Insight, Inc., Tokyo, Japan https://in.m.aipsurveys.com). The survey agency used a computer algorithm for the random sampling method. We defined chronic low back pain as low back pain lasting 3 months or longer (unrelated to pain intensity) and chronic daily headache as [26] a headache frequency of 15 days or more per month for 3 months (unrelated to pain intensity) [27]. Of the 600 study participants, 50 participants with chronic back pain and 50 participants with chronic headache completed the PDI-J 7 days after the first administration to assess test–retest reliability. More specific information about the DC-JBAP2020, along with a detailed description of the method used for recruiting participants, is provided in S1 Methods and S1 Fig: Participant Enrolment Process. Before responding to the online self-report questionnaire, we obtained web-based written informed consent from all participants.

### Measures

#### PDI-J

The original PDI assesses pain-related interference with a range of daily activities [5, 8]. The item content of the PDI reflects the definition of “disability” according to the Institute of Medicine Committee on Pain, Disability, and Chronic Illness Behavior, that is, “a disadvantage for an individual (resulting from an impairment or functional limitation) that limits or prevents the fulfillment of a role that is normal for that individual [28].” Pollard [5] developed the original version and then published its preliminary validity study in 1984. In 1994, the instructional text of the original version of PDI was slightly revised, but it retained the seven original items [8].

We developed a linguistically valid Japanese version of the PDI (PDI-J) and the PDI-5-J translated from the revised version of the PDI according to the guidelines for the translation and cultural adaptation of patient-reported outcome measures established by the task force of the International Society for Pharmacoeconomics and Outcomes Research [25, 29]. First, the PDI was translated from English to Japanese (A.M. and T.N.) and then harmonized (K.Y.). Next, a trilingual speaker of Japanese, English, and French (S.K.) back-translated the PDI from Japanese to English. An expert in self-report metholodogy for the assessment of disability reviewed the back-translation. The detailed procedure of the translation has been described elsewhere [25]. The linguistically validated PDI-J and PDI-5-J are shown in S2 and S3 Figs.

Similar to the original version, the PDI-J assesses pain interference with seven domains of daily activities: (1) Family/Home Responsibilities, (2) Recreation, (3) Social Activity, (4) Occupation, (5) Sexual Behavior, (6) Self-care, and (7) Life-support Activities [5, 8]. Respondents rate their level of disability for each domain on 11-point scales ranging from 0 (no disability) to 10 (total disability). Respondents’ scores on each item are totaled to obtain the overall score, which can range from 0 to 70 points.

#### PDI-5-J

In our previous pilot study on the adaptation of the PDI-J [25], some participants had expressed discomfort responding the Sexual Behavior and Life-support Activities items. In consideration of comfort in responding and accessibility to the Japanese population, we excluded the Sexual Behavior and Life-support Activities items from the PDI-J. Japanese people generally feel hesitant answering questions about sexual behavior, and are uncomfortable admitting to limitations that might be interpreted as an imposition on others. The PDI-5-J assesses pain interference with five domains of daily activities: (1) Family/Home Responsibilities, (2) Recreation, (3) Social Activity, (4) Occupation, and (5) Self-care. Respondents’ scores on each item are summed to obtain the overall score, which can range from 0 to 50 points.

#### Pain Disability Assessment Scale

The Pain Disability Assessment Scale (PDAS) assesses pain interference with daily activities (i.e., *physical function*) in the past week [30]. Our respondents rated their current pain interference according to 20 activities with the following anchors: (0) pain did not interfere with this activity, (1) pain interfered partially with this activity, (2) pain interfered significantly with this activity, and (3) pain interfered completely with this activity; the scores ranged from 0 to 60 [30]. In the present dataset, Cronbach’s alphas of PDAS were 0.93 in chronic low back pain and 0.97 in chronic daily headache.

#### Pain intensity

Pain intensity in the past week was measured by a single item numerical rating scale (NRS), ranging from 0 (no pain at all) and 10 (excruciating pain) [31].

#### McGill Pain Questionnaire–Short Form– 2

Pain quality in the past week was assessed using the SF-MPQ-2 [32, 33]. Participants rated the severity of their pain quality by 22 expressions (i.e., items), ranging from 0 (none) to 10 (worst possible); the overall score was 0–220 [32, 33]. In the present dataset, Cronbach’s alphas of PDAS were 0.94 in chronic low back pain and 0.95 in chronic daily headache.

#### Demographic information

We collected the following demographic information: age, sex, marital status, and educational attainment.

### Procedure and statistical analysis

All statistical procedures for examining the psychometric analyses of PDI-J and PDI-5-J were conducted separately for participants with chronic low back pain and chronic daily headache. Normality of PDI-J and PDI-5-J were assessed by Kalmogorov-Smirnov test.

Factor structure (i.e., dimensionality) was first determined by exploratory factor analysis (EFA) using promax rotation and the maximum likelihood estimation method. Confirmatory factor analyses (CFA) were then conducted to confirm the factor structure of the PDI-J and the PDI-5-J. We also evaluated a modified model using covariance error terms, following a modified index (MI). We considered MI >10.83 as significant [34]. In the PDI-J among participants with chronic low back pain, MI between item 2 and item 5 was 12.9, item 2 and item 7 was 10.2, and item 6 and item 7 was 46.7; that among participants with chronic daily headache, MI between item 2 and item 3 was 43.3, item 2 and item 6 was 15.7, item 2 and item 7 was 34.1, and item 6 and item 7 was 36.0. In addition, we considered both item 6 (Self Care such as taking a shower, driving, and getting dressed) and 7 (Life-Support Activities such as eating, sleeping, and breathing) as assessing physical function rather than social role, as well, the Japanese adaptation of these items reflects substantial semantic overlap. Therefore, we set a covariance error path between item 6 and item 7 for PDI-J among chronic low back pain and chronic daily headache. Similarly, we considered both item 2 (Recreation) and 5 (Sexual Behavior) as assessing pleasurable life activities, and both item 2 (Recreation) and item 3 (Social Activity) as assessing activities for leisure rather than activities for duty. As well, the Japanese adaptation of these items reflects substantial semantic overlap. Common or shared functional abilities required to perform recreational activities (item 2), life support activities (item 7), and self-care activities (item 6). Therefore, we also set a covariance error path between item 2 and item 5 among low back pain, and between item 2 and item 3, between item 2 and item 7, and between item 2 and item 6 among headache.

Model 1 was a one-factor model without an error covariance term; Model 2 was also a one-factor model but with one error covariance term between item 6 and item 7; Model 3 was added one error covariance term between 2 and 5 to Model 2. Model 4 was a one-factor model but with one error covariance term between item 2 and item 3; Model 5 added one error covariance term between 6 and 7 to Model 4; Model 6 added one error covariance term between 2 and 7 to Model 5; Model 7 added one error covariance term between 2 and 6 to Model 6; and Model 8 added one error covariance term between 2 and 6 to Model 4. For participants with chronic low back pain, Model 1, Model 2, and Model 3 were used in PDI-J, and Model 1 was used in PDI-5-J. For participants with chronic daily headache, Model 1, Model 4, Model 5, Model 6, and Model 7 were used in PDI-J, and Model 1, Model 4, and Model 8 were used in PDI-5-J.

Furthermore, we used *χ*^2^, *χ*^2^/*df*, root mean square error of approximation (RMSEA), and the standardized root mean square residual (SRMR) as fit indices. We used the following general cutoff points for the RMSEA: <0.05 (excellent fit), <0.08 (moderate fit), and ≥0.08 (poor fit) [35]. The SRMR values of ≤0.08 indicated good fit [36]. The SRMR values of ≤0.08 indicated good fit [37]. The comparative fit indices (CFI) and Tucker–Lewis index (TLI) were used as relative fit indices. The CFI and TLI compare the fit of a tested model with that of an alternative model (i.e., a model with the worst fit) [38]. The CFI and TLI values of >0.90 or 0.95 also indicated good fit [37, 39–41]. In addition, concurrent validity was evaluated using Pearson’s correlations among the PDI-J, PDI-5-J, PDAS, NRS, and SF-MPQ-2. The correlation coefficients which are ≤ 0.35 are considered as low correlations, 0.36 to 0.67 moderate correlations, and 0.68 to 1.0 high correlations with ≥ 0.90 very high correlations [42].

In assessing PDI-J and PDI-5-J reliability, internal consistency was assessed by Cronbach’s α, and test–retest reliability was evaluated by intraclass correlation (ICC). Cronbach’s alpha coefficient ranges between 0 and 1; and the value of Cronbach’s alpha ≥ 0.9 indicates excellent, ≥ 0.8 indicates good, ≥ 0.7 indicates acceptable, ≥ 0.6 indicates questionable, ≥ 0.5 indicates poor, and ≤ 0.5 indicates unacceptable [43]. ICC values < 0.5 are poor reliability, values 0.5 to 0.75 are moderate reliability, values 0.75 to 0.90 are good reliability, and values ≥ 0.90 are excellent reliability [44].

Moreover, *p* values of <0.05 (two-tailed tests) were considered statistically significant. We used IBM SPSS Amos version 23 (IBM Corp., New York, USA) for CFA, and IBM SPSS version 24 (IBM Corp., New York, USA) for calculating ICC coefficient using a two-way random effect model. Other statistical data were analyzed using SAS version 9.4 (SAS Institute Inc., Cary, NC, USA).

### Sample size for the factor analysis

This research recruited 300 participants with chronic low back pain and 300 participants with chronic daily headache. Several sample size guidelines have been suggested for factor analyses. For example, there is the criterion based on the total sample size: such that sample sizes of 300 or more are considered as good, and sample sizes of 1000 or more are considered excellent [45]. The current sample of 300 participants per group would be considered good following this criterion. There are also guidelines based on a ratio of observations to the number of items included in the factor analysis [46–50]. In the present study, the ratio of observations was to items was 20:1 which would be considered acceptable by several writers in this area.

### Ethical concerns

All procedures conformed to the ethical standards of the 1975 Declaration of Helsinki (revised in 2013). The Ethics Committee at Juntendo University Faculty of Medicine approved this study (approval date 15 October 2020; approval number 2020175). All participants provided their web-based informed consent before responding to the online questionnaire for DC-JBAP2020.

## Results

### Participant characteristics

Table 1 shows the participants’ characteristics.

**Table 1:** Mean and proportion of characteristics (n = 600).

	Low back pain		Headache		
	**Mean**	**SD**	**Mean**	**SD**	
**Age, years**	51.4	8.2	46.7	8.9	
	**n**	**%**	**n**	**%**	
**Sex**					
** Men**	227	75.7	154	51.3	
** Women**	73	24.3	146	48.7	
**Marital status**					
** Married**	211	70.3	172	57.3	
** Single**	54	18.0	83	27.7	
** Divorced**	27	9.0	39	13.0	
** Widowed**	8	2.7	6	2.0	
**Educational attainment**					-
** Less than high school**	9	3.0	16	5.3	
** High school**	62	20.7	74	24.7	
** Vocational school**	37	12.3	41	13.7	
** Junior college**	22	7.3	36	12.0	
** College of technology**	4	1.3	2	0.7	
** University**	148	49.3	107	35.7	
** Graduate school**	18	6.0	23	7.7	
** Other**	0	0.0	1	0.3	
	**Mean**	**SD**	**Mean**	**SD**	***P***
**Scores of scales**					
** PDI-J: 0–70**	19.9	13.2	26.2	15.9	***
** PDI-5-J: 0–50**	15.6	10.1	19.9	11.7	***
** PDAS: 0–60**	13.1	8.8	16.6	13.3	***
** NRS: 0–10**	4.7	1.9	4.9	2.0	
** SF-MPQ-2: 0–220**	40.3	35.4	50.1	42.4	**

*Abbreviation*: NRS, numerical rating scale; PDI-J, Japanese version of the Pain Disability Index; PDI-5-J, Japanese version of the 5-item Pain Disability Index; SD; standard deviation, SF-MPQ-2; Revised version of the Short-Form McGill Pain Questionnaire.

**p <0.01

***p <0.001.

The majority of participants were married and had completed at least 12 years of schooling. Based on scores in the MPQ-SF and the PDAS, the study sample would be characterized as experiencing moderate levels of pain and mild to moderate levels of disability.

### Scale characteristics

The mean scores of the PDI-J and PDI-5-J were 19.9 (standard deviation [SD], 13.2) and 15.6 (SD, 10.1) in chronic low back pain, and 26.2 (SD, 15.9) and 19.9 (SD, 11.7) in the chronic daily headache sample, respectively. The scores of the PDI-J, PDI-5-J, PDAS, and SF-MPQ-2 were higher in the chronic daily headache group than in the chronic low back pain group. Meanwhile, the NRS score was not different between the two pain samples. The distributions of the PDI-J and PDI-5-J scores are shown in S4 and S5 Figs. These are parabolic, as opposed to normal distributions other than the PDI-J scores among participants with chronic headache. P-values calculated by Kalmogorov-Smirnov test were <0.001 for the PDI-J and the PDI-5-J among participants with chronic low back pain, and 0.08 for the PDI-J and <0.001 for the PDI-5-J scores of participants with chronic headache.

### Validity

**Fig 1:**
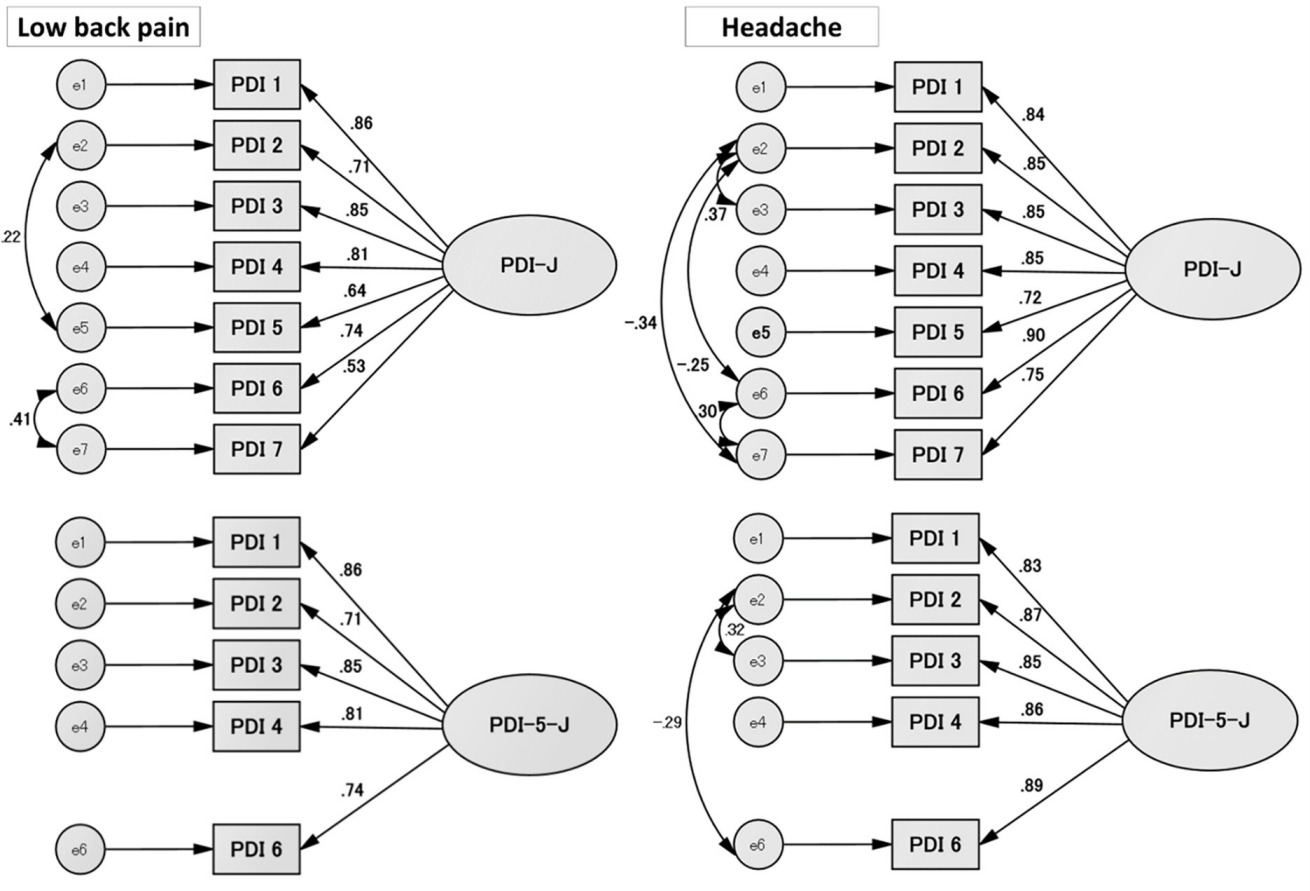
Final path diagrams of one-factor models for the PDI-J and PDI-5-J in chronic low back pain and chronic daily headache. One-factor models of the PDI-J and PDI-5-J with factor loadings and error terms (e1-e7 in PDI-J and e1-e5 in PDI-5-J) are indicated. The left side represents the chronic low back pain, and the right side represents the chronic daily headache. *Abbreviation*: PDI-J; Japanese version of the Pain Disability Index, PDI-5-J; Japanese version of the 5-item Pain Disability Index.

One factor was confirmed by EFA in the PDI-J and the PDI-5-J among patients with chronic low back pain and those with chronic dairy headache. Fig 1 shows the path diagrams of one-factor models for the PDI-J and PDI-5-J among the chronic low back pain and chronic daily headache samples. All factor loadings showed a significantly high score (>0.40).

Table 2 shows the goodness-of-fit summary for one-factor solution.

**Table 2:** Goodness-of-fit indices (one-factor solution) for the confirmatory factor analyses of PDI-J and PDI-5-J.

	*χ*^2^(*df*)	*χ*^2^/*df*	RMSEA (90% CI)	SRMR	CFI	TLI
**Low back pain**						
** PDI-J**						
** **Model 1: one factor (7 items)	83.92 (14)	5.99	0.13 (0.10, 0.16)	0.049	0.94	0.91
** **Model 2: one factor (7 items) + 1 error covariance between item 6 and 7	34.16 (13)	2.63	0.07 (0.04, 0.10)	0.03	0.98	0.97
** **Model 3: one factor (7 items) + 2 error covariance between item 6 and 7, and between item 2 and 5	22.12 (12)	1.84	0.05 (0.01, 0.08)	0.02	0.99	0.99
** PDI-5-J**						
** **Model 1: one factor (5 items)	11.56 (5)	2.31	0.07 (0.02, 0.12)	0.02	0.99	0.99
**Headache**						
** PDI-J**						
** **Model 1: one factor (7 items)	125.78 (14)	8.98	0.16 (0.14, 0.19)	0.04	0.94	0.90
** **Model 4: one factor (7 items) + 1 error covariance between item 2 and 3	74.87 (13)	5.76	0.13 (0.10, 0.16)	0.03	0.96	0.94
** **Model 5: one factor (7 items) + 2 error covariance between item 2 and 3, and between item 6 and 7	49.53 (12)	4.13	0.10 (0.07, 0.13)	0.02	0.98	0.96
** **Model 6: one factor (7 items) + 3 error covariance between item 2 and 3, between item 6 and 7, between item 2 and 7	29.47 (11)	2.68	0.08 (0.04, 0.11)	0.02	0.99	0.98
** **Model 7: one factor (7 items) + 4 error covariance between item 2 and 3, between item 6 and 7, between item 2 and 7, and between item 2 and 6	19.87 (10)	1.99	0.06 (0.02, 0.09)	0.02	0.99	0.99
** PDI-5-J**						
** **Model 1: one factor (5 items)	37.54 (5)	7.51	0.15 (0.11, 0.19)	0.02	0.97	0.95
** **Model 4: one factor (5 items) + 1 error covariance between item 2 and 3	12.11 (4)	3.03	0.08 (0.03, 0.14)	0.01	0.99	0.98
** **Model 8: one factor (5 items) + 1 error covariance between item 2 and 6	0.84 (3)	0.28	<0.001 (0.00–0.06)	0.003	1.00	1.00

*Abbreviation*: CFI, comparative fit indices; CI, confidence intervals; *df*, degrees of freedom; RMSEA, root mean square error of approximation; SRMR, standardized root mean square residual; TLI, Tucker–Lewis Index; *χ*^2^, chi square.

For Model 1 and Model 2 in chronic low back pain, the SRMR, CFI, and TLI suggested that the PDI-J showed a good fit but the RMSEA indicated otherwise. After an error covariance (Model 3), the RMSEA indicated a goodness-of-fit. Additionally, Model 1 of the PDI-5-J showed a good fit by SRMR, CFI, and TLI, and a goodness-of-fit by RMSEA.

In chronic daily headache, the SRMR and CFI for Model 1 suggested that the PDI-J indicated a good fit, but the TLI and RMSEA indicated slightly short of the score for a good fit. In Model 4, the TLI was improved, whereas the RMSEA remained the same. After four error covariance (Model 7), the RMSEA indicated a good fit. For Model 1 of the PDI-5-J, the SRMR, CFI, and TLI suggested a good fit, but the RMSEA suggested slightly short of the score for an adequate fit. After two error covariance (Model 8), the RMSEA indicated an adequate fit.

Table 3 shows Pearson’s correlations coefficient to demonstrate concurrent validity.

**Table 3:** Pearson’s correlations coefficient (95% confidence interval).

	PDI-J	PDI-5-J	PDAS	NRS
**Low back pain, n = 300**				
** PDI-5-J**	0.98 (0.97, 0.98)***			
** PDAS**	0.60 (0.52, 0.67)***	0.57 (0.49, 0.64)***		
** NRS**	0.40 (0.52, 0.67)***	0.38 (0.28, 0.48)***	0.36 (0.25, 0.45)***	
** SF-MPQ-2**	0.61 (0.53, 0.67)***	0.56 (0.48, 0.63)***	0.46 (0.37, 0.55)***	0.47 (0.37, 0.55)***
**Headache, n = 300**				
** PDI-5-J**	0.98 (0.98, 0.99)***			
** PDAS**	0.40 (0.30, 0.49)***	0.39 (0.28, 0.48)***		
** NRS**	0.44 (0.34, 0.53)***	0.41 (0.31, 0.50)***	0.25 (0.14, 0.35)***	
** SF-MPQ-2**	0.51 (0.42, 0.59)***	0.46 (0.36, 0.54)***	0.32 (0.21, 0.42)***	0.51 (0.42, 0.59)***

*Abbreviation*: NRS, numerical rating scale; PDAS, Pain Disability Assessment Scale; PDI-J, Japanese version of the Pain Disability Index; PDI-5-J, Japanese version of the 5-item Pain Disability Index, SD; standard deviation.

*** *p* < 0.001

Correlation coefficients of the PDI-J and PDI-5-J scores in chronic low back pain and chronic daily headache were both 0.98. Those of the PDI-J score and the PDAS, NRS, and SF-MPQ-2 scores in chronic low back pain and chronic daily headache were 0.40–0.61 and 0.40–0.51, respectively. In addition, those of the PDI-5-J score and the PDAS, NRS, and SF-MPQ-2 scores in chronic low back pain and chronic daily headache were 0.38–0.57 and 0.39–0.46, respectively.

### Reliability

The Cronbach’s α results of the PDI-J score in chronic low back pain and chronic daily headache were both 0.89. Those of the PDI-5-J score were 0.94 and 0.93 in chronic low back pain and chronic daily headache, respectively. The ICC coefficients of the PDI-J score for chronic low back pain and chronic daily headache were 0.67 (95% CI, 0.48–0.80; *p* < 0.001) and 0.59 (95% CI, 0.37–0.74; *p* < 0.001), respectively. Those of the PDI-5-J score for chronic low back pain and chronic daily headache were 0.57 (95% CI, 0.35–0.73; *p* < 0.001) and 0.63 (95% CI, 0.43–0.77; *p* < 0.001), respectively.

## Discussion

The results of this study supported the psychometric validity and reliability of the PDI-J and PDI-5-J tested on Japanese workers with chronic low back pain and chronic daily headache.

### Validity

The linguistically valid PDI-J and PDI-5-J used in this study attained the cross-cultural validity, in correspondence with the international recommendation. EFA supported a one-factor solution in the PDI-J and PDI-5-J. Goodness-of-fit indices for participants with chronic low back pain and chronic daily headache suggested a good fit.

The PDI-J and PDI-5-J scores were highly correlated (r = 0.98 in both pain conditions). Pearson’s correlation coefficients showed a moderate correlation between the PDAS and PDI-J or PDI-5-J. In addition, the PDI-J and PDI-5-J scores moderately correlated with the NRS and SF-MPQ-2 scores. These results are consistent with previous studies that examined the correlation between pain intensity and PDI score [10, 51, 52].

### Reliability

Cronbach’s α showed high internal consistency in the PDI-J or PDI-5-J, consistent with the results in previous studies using the original PDI [7, 8, 52, 53]. The test–retest reliability of both the PDI-J and PDI-5-J was acceptable, but the ICC coefficients were slightly lower than those in a previous study [53]. In the present study, the participants answered the second survey a week after the first survey. Although the PDI-J did not ask the period of their disability, the participants’ pain symptoms may slightly fluctuate in a week.

### Limitations

There are limitations that should be considered when interpreting the findings. Data for this study were obtained from a web-based study. There are indications that respondents to web-based surveys might differ in important ways from members of the general population. Additionally, the sampling issues with web-based surveys have been described previously [54]. These procedural factors necessarily have implications for the representativeness of the study samples and, relatedly, the generalizability of the findings.

## Conclusions

The present study provides evidence that the PDI-J and PDI-5-J demonstrated sound psychometric properties among Japanese workers with chronic pain, including chronic low back pain and chronic daily headache. Therefore, both the PDI-J and PDI-5-J may be useful tools for evaluating pain-related interference with daily life activities among the Japanese general population, accounting for role functioning.

## Supporting information

S1 Methods Specific information about the Data-Collection for Japanese Biopsychosocial Assessment of Pain in 2020 (DC-JBAP2020), along with a detailed description of the method used for recruiting participants. (PDF)

S1 Fig Participant enrolment process. (TIF)

S2 Fig The linguistically validated PDI-J. (TIF)

S3 Fig The linguistically validated PDI-5-J. (TIF)

S4 Fig The distributions of the PDI-J score. (TIF)

S5 Fig The distributions of the PDI-5-J score. (TIF)

S1 File AMOS_Backpain: The dataset (low back pain) for AMOS. (CSV)

S2 File AMOS_Headache: The dataset (headache) for AMOS. (CSV)

S3 File SAS and SPSS: The dataset for SAS and SPSS. (CSV)
