# Supplementary material for: Reliability and validity of the Japanese version of Pain Disability Index
Source: PLoS One. 2022 Sep 12;17(9):e0274445. doi: 10.1371/journal.pone.0274445 (PMC9467349; doi:10.1371/journal.pone.0274445)
Supplement: S1 Methods — (PDF) [file pone.0274445.s001.pdf]

S1_Supplementary Methods

Supplementary Methods: Internet Survey

Participants included in the Data-Collection for Japanese Biopsychosocial Assessment of Pain in 2020 (DC-JBAP2020) were recruited via e-mail invitation from panelists who had registered and specified having low back pain (n = 12,521) or headache (n = 15,596) with a Japanese internet survey agency (Rakuten Insight, Inc., Tokyo, Japan https://in.m.aipsurveys.com). Approximately 2.2 million panelists were registered in the survey agency and labeled in advance for specific purposes (e.g., having symptoms, expectant mother, and students). The survey agency has previously described their methods of quality control for the sampling of panelists [1]. Respondents to the web-based questionnaire agreed to provide web-based written informed consent and intended to participate in the survey for the DC-JBAP2020.

Response rate

The American Association for Public Opinion Research defines the response rate as “the number of complete interviews with reporting units divided by the number of eligible reporting units in the sample [2].” The internet survey agency was unable to determine whether each candidate received and/or recognized the invitation e-mail or not. Therefore, only the final number of participants in the survey was available. In the DC-JBAP2020, the response rate was defined as the proportion of the number of respondents relative to the number of e-mail invitations. A total of 12,500 invitations (6,250 for low back pain and 6,250 for headache) were delivered, and 5,000 (2,500 with low back pain and 2,500 with headache) people responded. Thus, the response rate in the DC-JBAP2020 was 5,000/12,500 (40.1%). The participation rate of internet surveys is likely to be lower than paper-based surveys administered on-site.

A detailed sampling method

The participant enrollment process is shown in Fig. S2. We requested the survey agency to recruit 300 workers aged 20–64 years with *chronic* low back pain and 300 workers aged 20–64 years with *chronic daily* headache using three screening questions described below. For secondary survey to examine test–retest reliability, we requested them to recruit 100 workers (50 workers with *chronic* low back pain and 50 workers with *chronic daily* headache) from these 300 workers, 1 week after the first recruitment.

The number of e-mail invitations (n = 12,500; 6,250 for low back pain and 6,250 for headache) and the minimum number of respondents (n = 5,000; 2,500 with low back pain and 2,500 with headache) to screen the target sample (300 workers with chronic low back pain and 300 workers with chronic daily headache) in the present internet survey was determined based on a statistical presumption by the survey agency.

S1_Supplementary Methods

The first invitation was sent to 12,500 candidates (6,250 for low back pain and 6,250 for headache) who were randomly selected from panelists with either low back pain (n = 12,521) or headache (n = 15,596) using a computer algorithm on October 16, 2020. Of the 5,000 respondents (2,500 with low back pain and 2,500 with headache) recruited for screening the target sample, the research agency selected 300 workers aged 20–64 years with *chronic* low back pain and 300 workers in the same age group with *chronic daily* headache using three screening questions. Subsequently, the second invitation was sent to all participants with *chronic* low back pain (n = 300) or *chronic daily* headache (n = 300) on October 23, 2020. Of these 600 participants, 118 participants (59 with *chronic* low back pain and 59 *chronic daily* headache) responded again (response rate, 50.9%). The survey agency randomly selected 50 workers with chronic low back pain and 50 workers with chronic daily headache for a secondary survey using a computer algorithm, following our request.

Questions for screening

The three questions used for screening were: (1) a question to detect a worker, (2) a question to detect having low back pain lasting ≥ 3 months in the past four weeks, and (3) a question to detect having headache occurs ≥ 15 days per month during 3 months.

* *Chronic* low back pain

(1) a worker and (2) having chronic low back pain, but NOT (3) having chronic daily headache

* *Chronic daily* headache

(1) a worker and (3) having chronic daily headache, but NOT (2) having chronic low back pain
