# Supplementary figures and images for: Reliability and validity of the Japanese version of Pain Disability Index
Source: PLoS One. 2022 Sep 12;17(9):e0274445. doi: 10.1371/journal.pone.0274445 (PMC9467349; doi:10.1371/journal.pone.0274445)

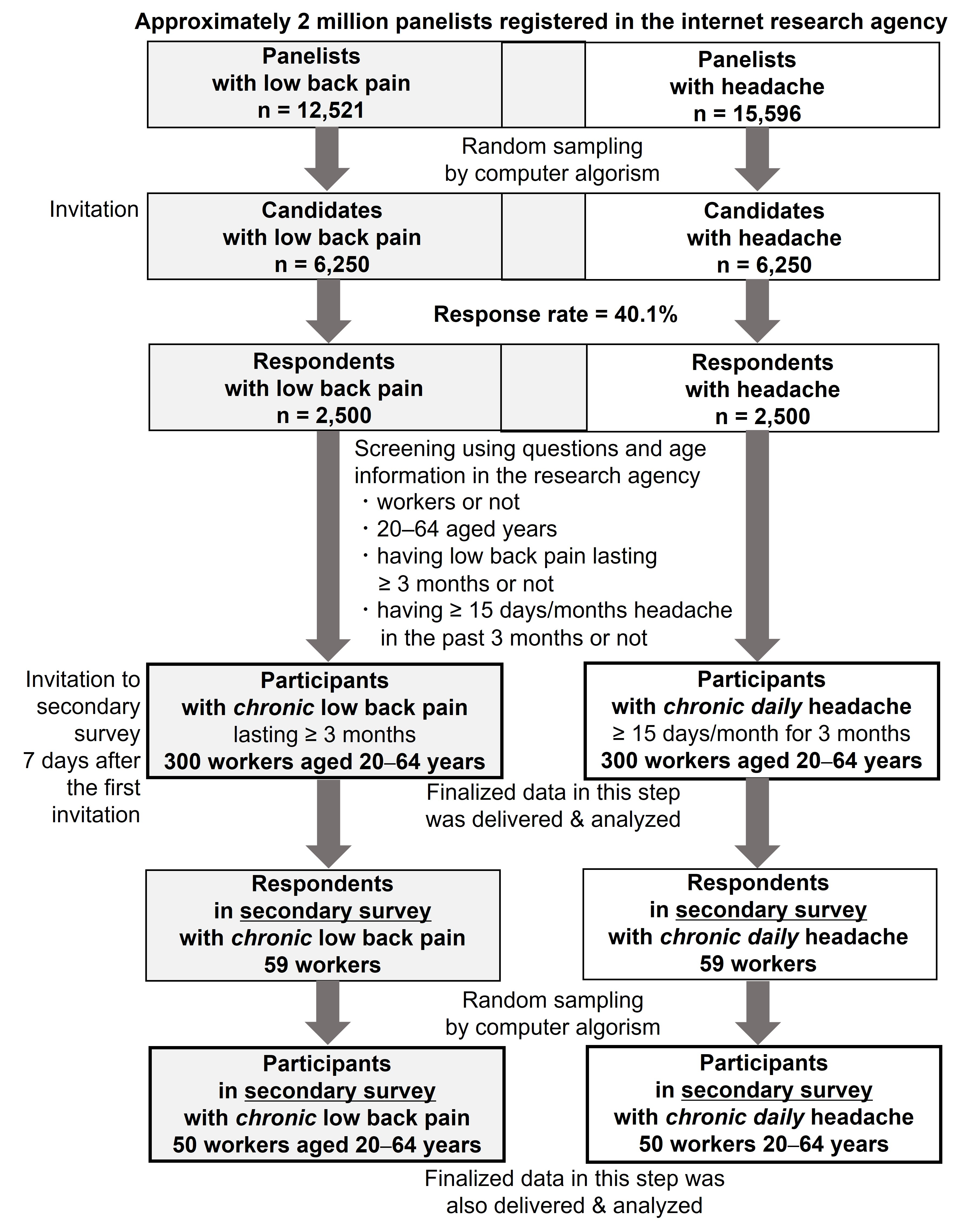

Supplement: S1 Fig — (TIF) [file pone.0274445.s002.tif]

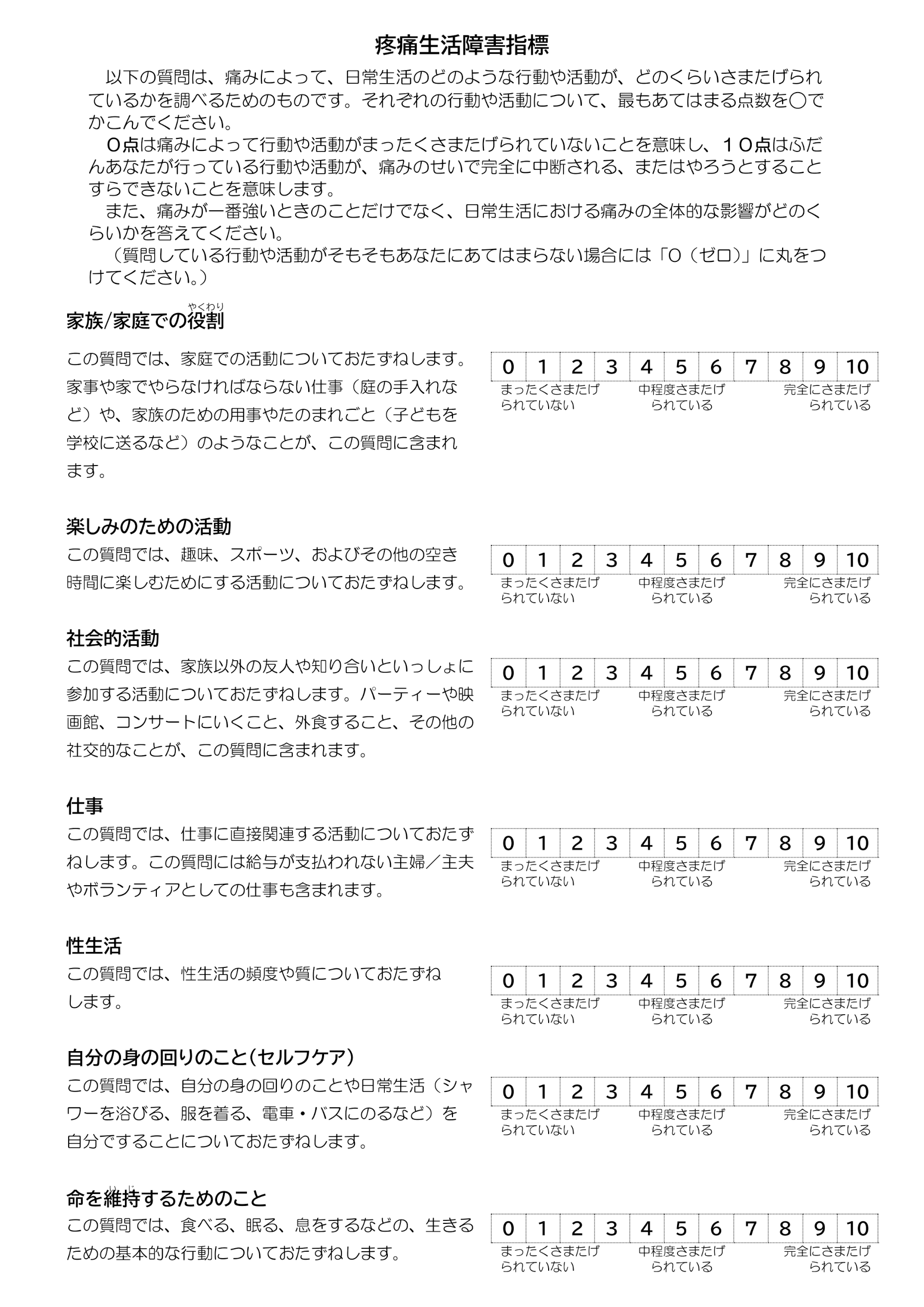

Supplement: S2 Fig — (TIF) [file pone.0274445.s003.tif]

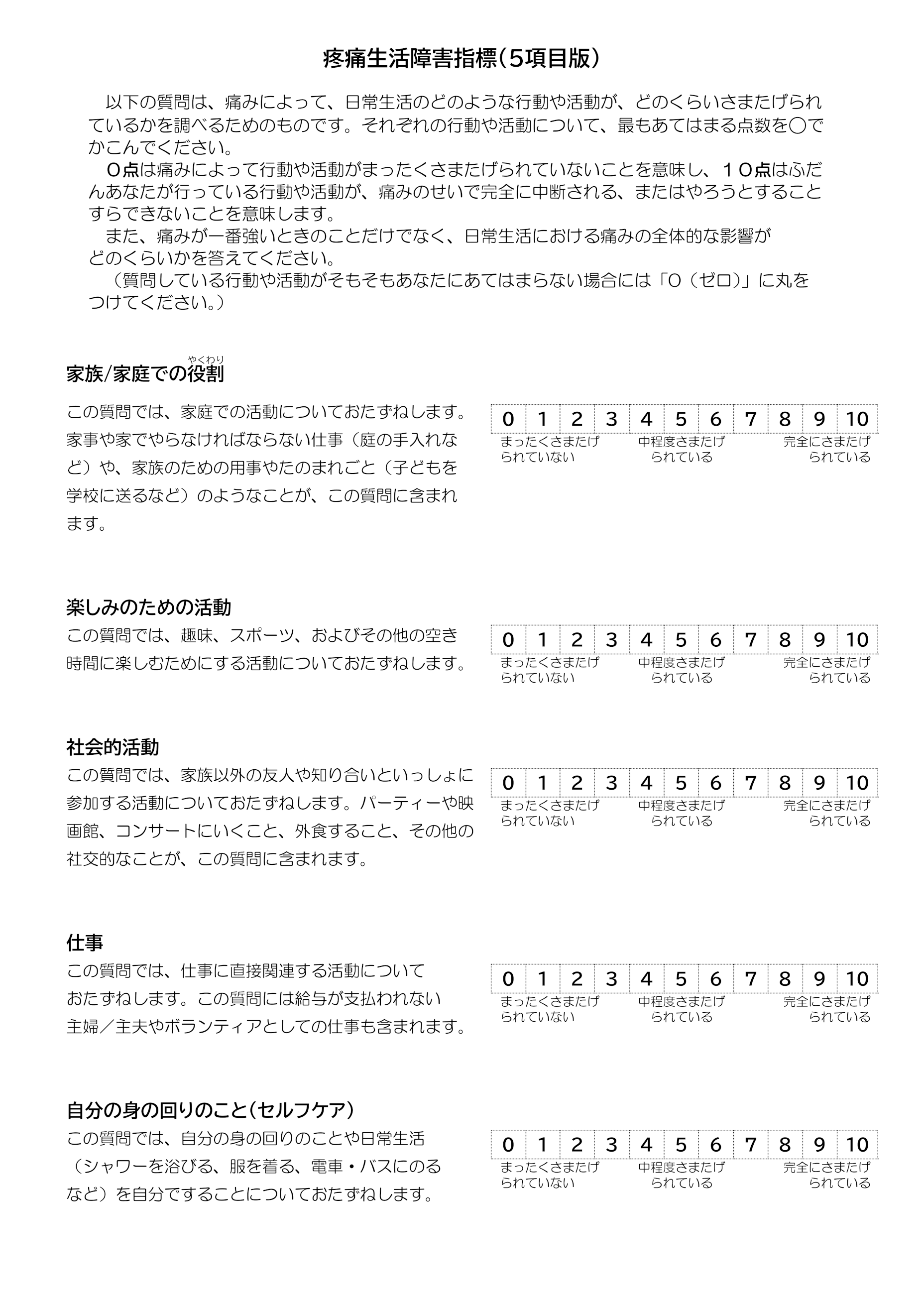

Supplement: S3 Fig — (TIF) [file pone.0274445.s004.tif]

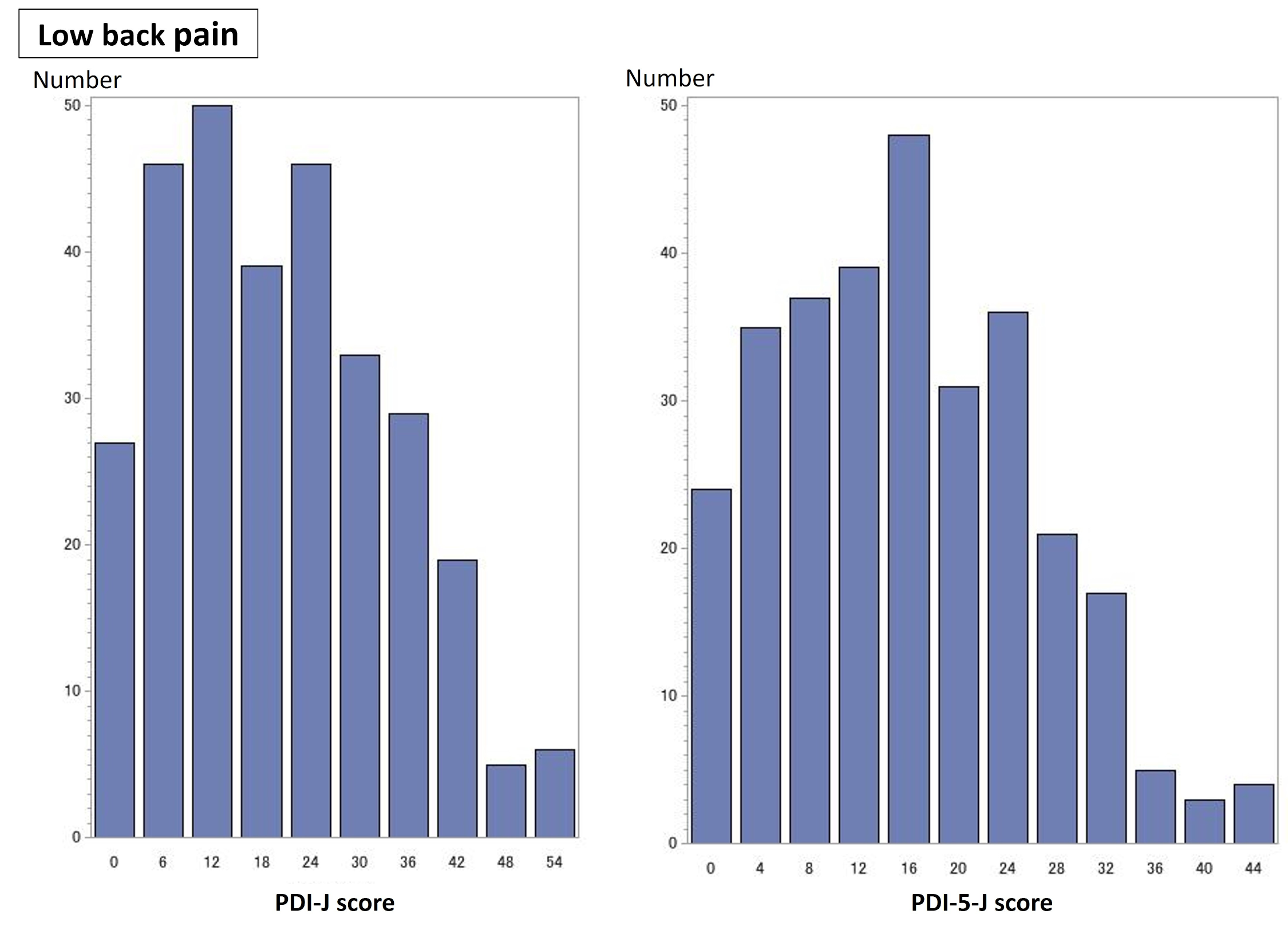

Supplement: S4 Fig — (TIF) [file pone.0274445.s005.tif]

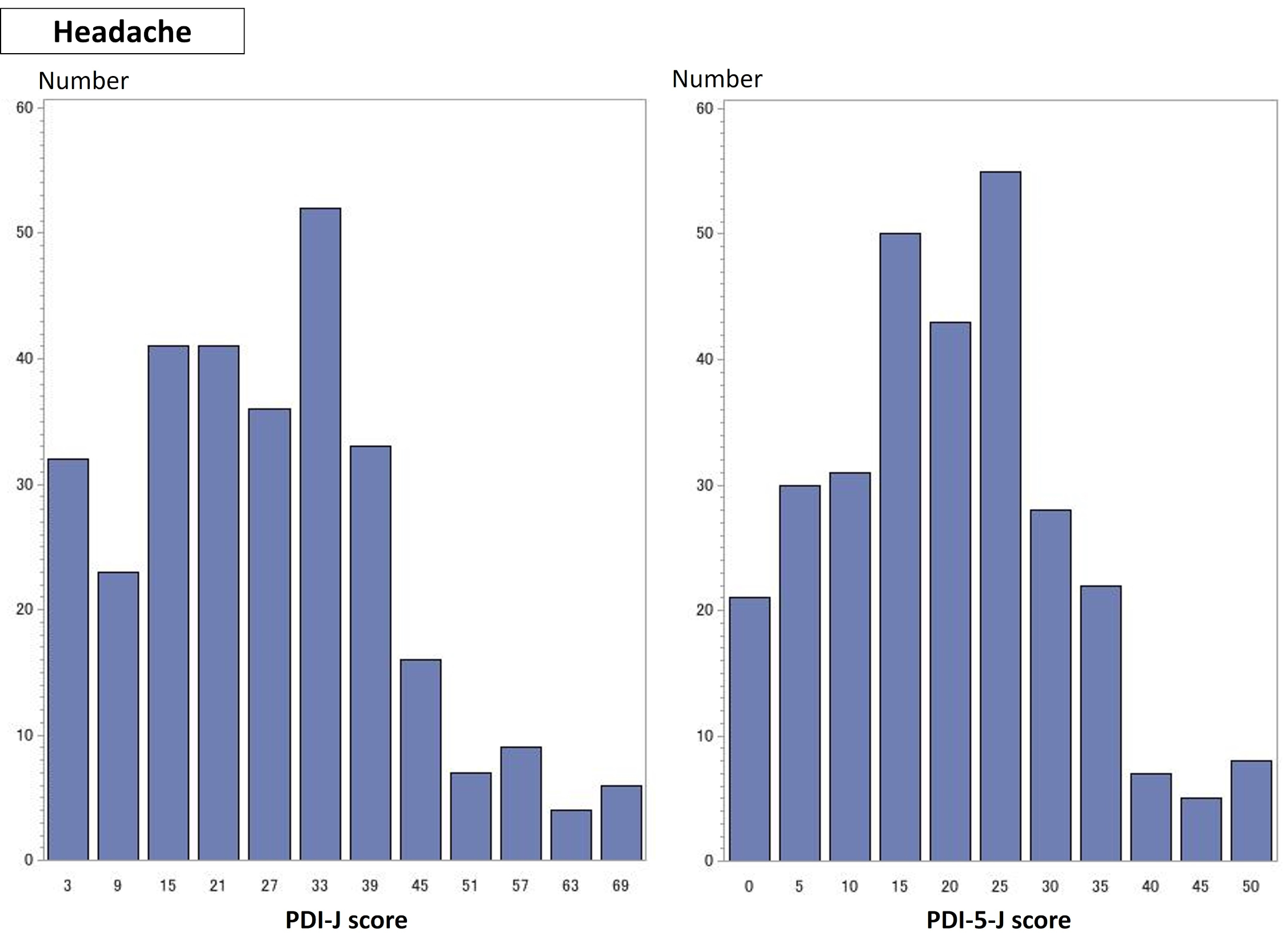

Supplement: S5 Fig — (TIF) [file pone.0274445.s006.tif]
